# Online Gambling Activity, Pay-to-Win Payments, Motivation to Gamble and Coping Strategies as Predictors of Gambling Disorder Among e-sports Bettors

**DOI:** 10.1007/s10899-021-10015-4

**Published:** 2021-03-10

**Authors:** Bernadeta Lelonek-Kuleta, Rafał Piotr Bartczuk

**Affiliations:** Institute of Psychology, The John Paul II Catholic University of Lublin, al. Racławickie 14, 20-950 Lublin, Poland

**Keywords:** Esports betting, Gambling disorder, Pay-to-win games, Coping, Gambling motivation

## Abstract

Research on esports activity usually captures it from the perspective of involvement in gaming. This study presents the results of the first research in Poland (*N* = 438) on esports betting (ESB). ESB is compared to other forms of e-gambling and involvement in pay-to-win games. The aim was to build a predictive model of gambling disorder among people betting on esports. A predictive model of gambling disorder based on ordinal regression was built, including sociodemographic variables, involvement in esports betting, involvement in other Internet activities connected to ESB, as well as psychological variables—motivation to gamble and coping strategies. The results showed that gambling disorder among esports bettors is associated with time spent on one game session, placing other forms of online gambling bets once a week or more often, and paying in pay-to-win games. Gambling disorder was also predicted by escape coping strategies and lower engaged strategies as well as financial and coping motivation to bet on esports results. The results show the crucial role of psychological factors (motivation, coping) in the development of esports betting addiction. Esports betting is an activity associated with both gambling and gaming—involvement in both activities explains the development of ESB addiction. There is a need for further research focused on the specificity of esports betting behavior to discover the direction of links among gaming, gambling, and esports gambling.

## Introduction

The gambling market has been relatively stable for decades, based on a finite pool of the main types of gambling available in specific locations (e.g., bars, hippodromes, casinos, and lottery outlets). The first significant changes in the gambling market were connected with the spread of access to the Internet and the accompanying gambling offerings. In the context of these changes, there was talk of a new phenomenon of online gambling, also referred to as e-gambling by analogy to other activities on the Internet, e.g., e-commerce or e-sport. E-gambling is gambling organized in the Internet space, where a person can participate via devices connected to the network (computer, mobile phone, tablet, etc.). This phenomenon has translated into a noticeable increase and changes in the nature of gambling involvement, which are visible in many countries. The reasons for these changes include anonymity, low cost, and ease of access, additional benefits, interactivity, game speed, and the possibility to play multiple games simultaneously (Barrault & Varescon, 2016; Choir, 2016; Gainsbury et al., 2012; Hing et al., 2016; McCormack et al., 2013). Although the results are not consistent, the research most often shows a higher percentage of problem gamblers among online gamblers than offline gamblers (Canale et al., 2016; Effertz et al., 2018; Williams et al., 2012). The same was observed in Poland, where the symptoms indicating probable gambling addiction have occurred in 26.8% of online gamblers (Lelonek-Kuleta et al., 2020), while in Polish studies covering offline and online gamblers, 11.8% have displayed a risk (low to high) of gambling addiction (Moskalewicz et al., 2019). The study by Chóliz (2016) showed that in 2 years from legalizing online gambling in Spain, there was a nearly tenfold (from 2.53 to 24.21%) increase in the frequency of indicating this form of gambling as the leading cause of problems by patients seeking treatment for gambling addiction. In a subsequent study, researchers noted a significant decline in young adults' interest (18–25) in offline games in favor of online games (Chóliz et al., 2019). In Poland, in 2011, online bookmakers were legalized as the only form of online gambling. Currently (2021), it is also possible to bet on esports bettings, lotteries, and gamble in an online casino.

Among the risk factors of online gambling addiction, apart from the features of online games themselves, researchers mentioned lower education, male gender, unemployment, and not being in a relationship (Effertz et al., 2018). Others pointed to the type and number of online gambling and the frequency of gambling, as related to problem online gambling (Gainsbury et al., 2015; McCormack et al., 2013). These factors distinguished offline and online gamblers and were more pronounced in the second group (Petry & Gonzalez-Ibanez, 2015).

The latest phenomenon in the gambling industry is the possibility of betting on the results of video games, i.e., esports. The phenomenon of esports itself is relatively new. It is a new type of gaming activity that is rapidly gaining in popularity and reach, especially among young players (Bányai et al., 2019; Griffiths, 2017; Marelić & Vukušić, 2019; Varga Szépné et al., 2019). Esports, or electronic sports, means professionalization of some gamers who start to treat gaming as a career and specialize in competing with other players (Bányai et al., 2019). These players define themselves as professional gamers or esports players and are different from casual players. A professional player plays more for competition than for entertainment or relaxation and treats the game as their job. A casual player plays for fun and recreation (Ma et al., 2013). The gaming industry (e.g., International Esports Federation, n.d.) now supports the opportunity to engage professionally in gaming competitions at large, through organizing tournaments with thousands of spectators, and sponsoring and offering prizes to winners on the order of thousands of dollars (Kim & Thomas, 2015). According to Whalen (2013), esports is "an umbrella term used to describe organized, sanctioned video game competitions, most often in the context of video game tournaments" (p. 23). There is a continuous discussion as to whether this form of recreation can be described as a sport at all. According to some authors, including this entertainment in the category of sport is legitimate (Adamus, 2012; Kane & Spradley, 2017; Vik-Hansen, 2020). However, some oppose such classification of this entertainment (Abanazir, 2019; Frias & Triviño, 2016; Karhulahti, 2017; Llorens, 2017). According to Bányai et al. (2019), further work is needed to definitively assess whether or not esports is a real sport.

An important aspect of the new phenomenon of esports is linking it to gambling through the possibility of betting on the results of gaming competitions. The problem of similarities and the link between gaming and gambling has been addressed for a long time. For example, Griffiths wrote as early as 1991 about the multidimensional similarities between video games and slot machines (the occurrence of almost-wins, amplification mechanisms, structural characteristics of games, the effects of excessive playing). He described video games as a "non-financial form of gambling" (Griffiths, 1991, p. 54). Griffiths also drew attention to the analogy between professional gaming (or esports) and professional gambling, especially poker (Griffiths, 2017). Both types of games require certain skills, there are games and tournaments organized to attract large audiences, and the results of both games can be bet on. This option in ESB has brought e-gaming closer to gambling, making this aspect of gaming activity "pure" gambling. One of the options of betting on video game results is in-game items, i.e., attractive items in the game, and the space for betting is so-called social gaming, i.e., games that allow interaction between players (Grove & Krejcik, 2015; Macey & Hamari, 2018b). One type of social gaming is social casino gaming. These are freemium games that are not played for real money, but their atmosphere and the nature of entertainment are no different from real casinos (Gambling Commission, 2017). These games, due to the nonmonetary nature of the prizes (no money or monetary exchange value is involved), do not meet the criteria for gambling and are not regulated by law; furthermore, they are accessible to everyone, including children (Gainsbury et al., 2017b). According to the Gambling Commission (2017), in year 9% of children aged 11–15 years were involved in gambling without money, and 26% of them gambled with real money. Another point of contact between gaming and gambling is skin gambling. This is a special form of betting often offered by entities that are independent of game providers (publishers), and where the stakes are skins, which have an aesthetic value and do not, in principle, affect the course of the game (Greer et al., 2019). They are one of the types of virtual goods or items that usually have no real value outside the games. In some games, however, due to the possibility of selling them illegally on non-game sites, they have become prevalent and have become a kind of virtual currency, used, for example, in esports betting and other gambling (e.g., simplified versions of roulette) (Cleghorn & Griffiths, 2015; Grove, 2016b). In general, video gambling is accepted by adult players; however, all groups of game audiences and esports should be kept in mind.

The viewers of esports are also subject to researchers' interest (Adamus, 2012; Jenny et al., 2016). In light of the research results, the intensity of the view ratio of esports games is positively related to escape motivation (escapism) and the need to broaden one's knowledge about the games (Hamari & Sjöblom, 2017). There is an analogy to gaming disorder or gambling disorder, in which the escape motive plays a vital role (Chwaszcz et al., 2018; Eichenbaum et al., 2015; Hafeez et al., 2017). The willingness to learn about gambling, on the other hand, brings to mind the gambling-related distortions that make the player broaden his or her knowledge of gambling in the belief that this increases their ability to predict the outcome of the game (Armstrong et al., 2020). Experts point out the tendency of some players to overestimate the role of skills in betting, which is a particular risk factor for esports bettors (Bjerg, 2010; Owens, 2016; Zhou et al., 2012). For young people the possibility of winning a skin or other in-game item with a very high financial value in the illegal market can be a powerful temptation to gamble. The use of in-game items such as virtual currencies in gambling is becoming increasingly popular, and more and more gambling portals offer this possibility (Griffiths, 2017). For formal reasons, this form of betting is still not considered as gambling, but increasingly more attention is drawn to the fact that the opportunity to monetize virtual goods gives them real value and makes betting with their actual use gambling, as pointed out by the Gambling Commission (2017).

The great interest in esports and betting on its results have finally led many operators to include this activity in their offer of "real" gambling—the possibility of betting on video game results for real money (Esports Insider, 2018; Macey & Hamari, 2018a). Gambling operators also undertake the sponsorship of teams of sportsmen, which confirms the strong link between these activities (Holden et al., 2019). Due to the blurred boundaries between the various forms of betting on video games presented above, the transition from quasi-gambling (social casino gambling, skin gambling, etc.) to real gambling is very fluid, with the risks highlighted by researchers (Johnson & Brock, 2019; King, 2018). This risk is particularly acute for young people, who often come into contact for the first time with various forms of betting, including for real money (King, 2018).

According to a report by Narus Advisors and Eilers and Krejcik Gaming (Grove, 2016a), there is an increased propensity to gamble among esports fans. Furthermore, esports fans are twice as likely to gamble as average Internet users, while 60% use betting sites. Gainsbury et al. (2017b) drew attention to the need for further research on the risk of gamers betting on esports to further forms of traditional gambling and the consequent increase in the risk of gambling addiction. The Internet, as a medium, is a particular risk factor.

Despite the already undertaken research on esports betting and professional video gaming, Griffiths (2017) drew attention to the lack of knowledge in this area, both in the context of its regulation and the psychosocial effects of this activity. Greer et al. (2019) highlighted the scarcity of research on esports betting and the resulting lack of knowledge about the intensity of the phenomenon, esports bettors' characteristics, and their gambling behavior. One of the reasons for this deficit is the illegality of a large portion of this activity and limited access to data. One of the few studies on esports bettors was conducted in Australia (Gainsbury et al., 2017a, b).

There has been little research into gambling addiction among esports bettors. Gainsbury et al. (2017b) noted a higher risk of gambling addiction among gamers who practice both gaming and esports betting. The higher risk was related to esports bettors' perception of gambling as purely skill games based on an analogy to their skill-based video games. Gainsbury et al. (2017a) research showed that the severity of gambling disorders among esports bettors was higher than among sports bettors. Additionally, esports bettors have engaged in more and more gambling games with greater intensity than sports bettors. Research into the mechanisms of gambling addiction among esports bettors is limited. More often, research concerns esport gamers and problematic gaming (Bányai et al., 2019), although the authors also pay attention to this area's research deficit (Chung et al., 2019). Research on gambling addiction emphasizes the importance of poorer psychosocial functioning (Weinstock et al., 2012) and motivation (MacLaren et al., 2015; Reid et al., 2011). More typical for problem gamblers are financial motivations (Fortune & Goodie, 2012; Myrseth et al., 2010) and escaping from problems (Gentile et al., 2011; Loton et al., 2016). Additionally, a factor increasing the risk of gambling addiction is the intensity of involvement in gambling, which, interestingly, plays a smaller role among gamers (Dragicevic et al., 2011; Han et al., 2018).

The study presented in the present article aimed to identify variables predicting gambling addiction risk among e-sports bettors. As ESB is both gaming and gambling activity, these factors were sought after in both of these areas. Research to date has focused mainly on the search for a link between ESB and gaming—active gaming, both as a casual gamer and as a professional gamer, but also the viewing of esports games (spectator research) (Griffiths, 2017; Hamari & Sjöblom, 2017; Macey et al., 2020; Melbourne & Campbell, 2015). While new research aimed at explaining esports betting activity has begun to emerge (Macey et al., 2020), there are still very few studies on the explanation of ESB addiction (Gainsbury et al., 2017b), especially those that would take into account different forms of gaming activities (gaming, online gambling, and offline gambling).

Due to these gaps, we carried out this research on the factors that explain ESB addiction, which included gaming activity focused mainly on games with pay-to-win add-ons, which are closer to gambling than other games due to their financial aspects. Also, the analyses included gambling activity—different types of online and offline gambling and the use of virtual currencies in ESB, i.e., a currency used both for gambling and gaming.

## Methods

### Participants and Procedure

Esports bettors were selected from the broader sample of Internet gamblers. The survey was conducted by an IMAS Internet panel among registered users. The panel has operated in Poland since 2005, based on voluntary paid cooperation, following ESOMAR's research standards, and has over 65,000 registered employees (IMAS International Sp. z o.o., 2019). The respondents were rewarded for taking part in the study. IMAS invited 46,806 panel users to participate in the research. The invitation was accepted by 8511 (18.2%) of them. The respondents were selected based on two criteria: (1) they have gambled online during the last 12 months or (2) they have spent money on free-to-play games. Respondents meeting one or both of these criteria formed the research group of 2074 people. From this group, people who gave positive answers to the question: "Have you gambled for money on esports or virtual sports betting online over the past 12 months?" were selected. The esports bettors sample consisted of 438 people aged 18 to 64 (*M* = 33.1, *SD* = 9.27). Women constituted 37% and men 63% of respondents.

Other sociodemographic variables were also included in the study: education, monthly income, and marital/residence status. Table 1 presents the exact characteristics of the respondents in terms of sociodemographic variables.

**Table 1:** Education, income, and marital status of survey participants (N = 438)

Variable	Categories	n	%
Education	Below tertiary	204	46.6
	Tertiary	234	53.4
Monthly household net income	Up to PLN 2000	22	5.0
	PLN 2000–3999	98	22.4
	PLN 4000–5999	106	24.2
	PLN 6000–7999	101	23.1
	PLN 8000–11,999	59	13.5
	PLN 12,000–19,999	14	3.2
	PLN 20,000 and above	13	3.0
	Don't know	25	5.7
Cohabitation	Married or cohabitating	337	76.9
	Alone	101	23.1

PLN is Polish zloty abbreviation; 1€ ≈ 4.3 PLN

### Measures

#### Gambling Disorder

The intensity of gambling disorder among esports bettors was measured using the Polish translation of the Problem Gambling Severity Index (PGSI; Ferris et al., 2001). It contains nine statements to which the respondent gives answers on a 4-level response scale (0 = *Never*, 1 = *Sometimes*, 3 = *Most of the time*, 4 = *Almost always*). The interpretation of the results is as follows: 0 points—no gambling problem, 1–2—low gambling problem, 3–7—moderate gambling problem, 8 and more—problem player. The reliability of PGSI in the present study was α = 0.93. Results greater than or equal to 3 are included in the group of the pathological gamblers.

#### E-gambling

Information on online gambling was collected through a multiple-choice question, "From this list of games, in which one(s) have you gambled for money online over the past 12 months?" The list contained 11 types of Internet gambling games: 1. Lotteries of Totalizator Sportowy; 2. Other lotteries; 3. Scratch cards; 4. Slot machines; 5. Poker; 6. Other card games for money; 7. Other casino games; 8. Horse racing; 9. Sports betting (including fantasy sports); 10. Esports or virtual sports betting; 11. Betting on financial markets (FOREX, binary options).

#### Other Activities Connected to ESB

Additional potential online gaming activities included using virtual money for sports betting ("From this list of games, have you ever gambled online with in-game money over the past 12 months on each of the following activities?"), paying for pay-to-win games ("In the past 12 months, have you made payment?"), and gambling offline ("In the past 12 months, have you gambled offline (by going to a sale point, in a casino or on a racecourse…)?").

#### Motivation to Gamble

Gambling motivation was measured with the Polish translation of the Gambling Motives Questionnaire–Financial GMQ-F (Devos et al., 2017). The questionnaire comprises 16 questions to which the respondent gives answers on a 4-level response scale (1 = *Never or almost never*, 2 = *Sometimes*, 3 = *Often*, 4 = *Almost always*). The tool allows determining the intensity of the four motives of gambling: financial, social, enhancement, coping. In the present study, Cronbach's alpha reliability coefficients for subscales were as follows: enhancement, 0.79; social, 0.71; coping, 0.74; and financial, 0.80.

#### Coping Strategies

The Brief COPE questionnaire by Carver (1997) in the Polish adaptation of Juczyński and Ogińska-Bulik (Juczyński & Ogińska-Bulik, 2012) was used to measure stress coping strategies. It is composed of 28 statements comprising 14 larger units (two statements in each strategy). The respondent assesses the items on a 4-point scale from 0 (= *I haven't been doing this at all*) to 3 (= *I've been doing this a lot*). The method is most often used to measure dispositional coping, i.e., to assess typical responses and sensations in situations of severe stress. Because each strategy has only two items and the strategies correlate with each other, a dimensional reduction has been made here through principal component analysis (PCA) with varimax rotation. Both the scree test and the Kaiser criterion indicated two components to be extracted, which explained 53% of the variance of the Brief COPE subscale results. The results of the Brief COPE PCA are shown in Table 2.

**Table 2:** Exploratory principal component analysis of brief COPE subscales with varimax rotation—component loadings matrix (N = 438)

Subscale	PC1	PC2
Behavioural disengagement	**0.800**	− 0.256
Substance use	**0.755**	− 0.257
Denial	**0.744**	− 0.055
Venting	**0.688**	0.245
Religion	**0.676**	0.013
Self-blame	**0.663**	0.145
Humour	**0.642**	0.076
Self-distraction	**0.558**	0.325
Planning	− 0.182	**0.814**
Positive reframing	− 0.006	**0.796**
Active coping	− 0.259	**0.795**
Using emotional support	0.115	**0.736**
Using instrumental support	0.186	**0.714**
Acceptance	0.224	**0.621**

Component loadings larger than .4 are bolded

The first component was defined as an engaged coping style, including an active approach, seeking social support and cognitive mastery of the situation, and the second as an escape coping style, which included evasion countermeasures, denial, emotional response, and escape into psychoactive substances and religion.

### Statistical Analysis

Frequencies and percentages were used to describe categorical variables. Associations between predictors and psychological distress were assessed using cumulative link models (ordinal regression). Complementary log–log link function was used, reflecting the assumption that there is a high risk of problem gambling in the e-sports bettors' population. Multiple regression models were built to identify factors associated with the outcome variable, which was a 4-level problem gamblers classification based on PGSI (non-problem; low risk; moderate risk; problem). Potential predictors were grouped into five blocks, whose predictive power was then incrementally tested on pay-to-win with likelihood ratio tests. The final predictive model was built by the backward removal of non-contributing factors. The analysis also uses the property of the cumulative link models, which allows taking into account in the prediction modeling not only differences in the predictor level (location parameter) but also differences in its distribution (scale parameter).

### Ethics

The study procedures were carried out under the Declaration of Helsinki. In Poland, each university establishes its own requirements concerning obtaining ethics committee consent for research participation. At the [Name of the University], psychological surveys are directed to such committees only in special cases, depending on the decision of the person in charge of the research project section. In the case of voluntary online surveys regarding issues widespread in the population, such necessity was not reported. To comply with ethical standards, the research was conducted according to the standards of good research practice recommended by the American Psychological Association (APA). The participants were informed about the confidentiality and anonymity of the research, and that they had a right to resign from participation.

## Results

### Behavioral Characteristics of ESB

Among esports bettors, almost equal dominant groups emerged in terms of frequency of playing over the past 12 months—people playing several times a year (21.2%), several times a month (19.4%), several times a week (19.6%), and once a week (17.1%). Of the respondents, 11.6% played once a month, and 9.4% almost daily. Only 1.6% of the population were involved in esports betting every day. As for the amount of money spent on one ESB session, the respondents most often indicated PLN 10 (27.2%) and PLN 20 (22.1%); 15.5% spent less than PLN 5 and 8.9% PLN 50 for this purpose. The remaining subjects spent more money on one session, including 2.3% spending more than PLN 500. The largest group of players spent less than 15 min (29%) on a single e-sports betting session, and another group spent between 15 and 30 min (29.9%). Further, 24.4% of the players spent up to one hour on one gaming session, and 10.5% spent between one and two hours. Only 6.2% of the respondents spent more than 2 h on one game. Less than one-third of the players (30.8%) used virtual money in ESB.

### Prevalence of Gambling Disorder Among esports Bettors

Frequencies of four classification categories, based on the cut-points for PGSI, in the study group were as follows: non-problem gamblers (0 points in PGSI) accounted for 17.8% (*n* = 78, 95%CI[14.4%, 21.6%]); 19.6% (*n* = 86, 95%CI[16.1%, 23.5%]) showed a low risk of gambling addiction (1–2 points, low-risk gamblers); 27.6% (*n* = 121, 95%CI[23.6%, 32.0%]) showed a moderate level of addiction risk (3–7 points, moderate risk gamblers); while 34.9% (*n* = 153, 95%CI[30.6%, 39.5%]) esports gamblers exhibited gambling disorder symptoms (above 8 points in PGSI, problem gamblers).

### Other Forms of Gambling Among esports Bettors

Esports bettors were also asked about other types of gambling they have been involved in on the Internet over the past 12 months. Totalizator Sportowy state-run lotteries turned out to be the most popular, with 60.5% of the bettors admitting to participating in it. Next in line were scratch cards, sports betting, and online slot machines. Three-quarters of the esports bettors had been involved in another form of e-gambling once a week or more. Detailed results are shown in Table 3.

**Table 3:** Forms of online and offline gambling among esports bettors (N = 438) in the last 12 months before the study

Game	*n*	%
*Online gambling*		
1. TS lottery	265	60.5
2. Other lottery	154	35.2
3. Scratch cards	194	44.3
4. Slot machines	172	39.3
5. Poker	153	34.9
6. Other card games for money	86	19.6
7. Other casino games	78	17.8
8. Horse racing	66	15.1
9. Sports betting (including fantasy sports)	193	44.1
10. Betting on financial markets (FOREX, binary options)	116	26.5
Online gambling other than ESB at least once a week	333	76.0
*Offline gambling*		
1. TS lottery	159	63.9
2. Other lottery	44	17.7
3. Scratch cards	178	71.5
4. Slot machines	82	32.9
5. Poker	34	13.7
6. Other card games for money	16	6.4
7. Other casino games	7	2.8
8. Horse racing	39	15.7
9. Sports betting	134	53.8
10. Other games	2	0.8
Any form of offline gambling	249	56.8

The results show that 56.8% of the esports bettors had also been gambling outside the Internet within the 12 months before the survey. As far as offline gambling is concerned, scratch cards were the most popular, with 71.5% of players admitting to using them. Totalizator Sportowy lotteries and sports betting were next in line. The detailed results are contained in Table 3.

### Pay-to-Win Gaming Among esports Bettors

Analysis of the responses of esports bettors showed that 88.6% of them had also played free online games on their laptops, tablets, smartphones, or social media within the 12 months before the survey. In turn, 67.4% of the esports bettors had made payments in these games to purchase various add-ons. The vast majority of players paying in these games had paid to increase their chances of winning in such a free game. Of all bettors, 59.8% paid in free games to win.

### Prediction of Gambling Disorder Among esports Bettors

Ordinal regression was used to determine the factors that are predictors of gambling disorders among esports bettors. The variable was explained by four risk levels of gambling disorder obtained based on PGSI. Potential predictors were grouped into four blocks: 1. sociodemographics: gender (male/female), age (continuous), education level (higher/lower), cohabitation (alone/not alone), net income (continuous, midpoint recoded); 2. coping strategies: engaged and escape; 3. motivation to gamble: coping, enhancement, social, and financial; 4. behavioral features of ESB: frequency (ordinal with 7 categories), amount of money spent on ESB session (continuous, midpoint recoded), amount of time spent on ESB session (ordinal with 7 categories), using virtual money in ESB (dichotomous); and 5. other behaviors connected to ESB during last year: other e-gambling activities performed at least once a week (dichotomous), offline gambling during 12 months (dichotomous), making payments in pay-to-win games during 12 months (dichotomous).

The predictive power of blocks was incrementally tested to determine whether the subsequent variable groups—socioeconomic background, personality, motivation, ESB behavior, and other behaviors—explain gambling disorder among esports bettors. The models were compared with one another using the likelihood ratio test (cf. Table 4).

**Table 4:** Nested predictive models comparison (N = 438)

Variable	Block 1		Block 2		Block 3		Block 4		Block 5	
	*df*	χ^2^	*df*	χ^2^	*df*	χ^2^	*df*	χ^2^	*df*	χ^2^
Gender	1	1.46	1	0.41	1	0.11	1	0.02	1	0.00
Age	1	2.59	1	1.42	1	3.41	1	3.36	1	2.50
Education	1	1.77	1	1.43	1	0.12	1	0.04	1	0.00
Partner	1	0.19	1	0.05	1	0.05	1	0.01	1	0.11
Income	1	2.25	1	0.65	1	0.23	1	0.23	1	0.80
Escape coping			1	52.08***	1	22.81***	1	18.21***	1	16.55***
Engaged coping			1	5.08*	1	7.98**	1	4.99*	1	8.86**
Coping motive					1	42.79***	1	30.24***	1	25.15***
Enhancement motive					1	0.01	1	0.33	1	0.08
Social motive					1	0.07	1	0.18	1	0.20
Financial motive					1	4.85*	1	5.22*	1	5.14*
ESB frequency							6	9.49	6	6.49
ESB money							1	2.61	1	4.01*
ESB time							5	19.41**	5	15.26**
ESB virtual money							1	3.15	1	2.85
Other e-gambling									1	15.26***
Offline gambling									1	0.35
Play-to-win paying									1	7.30**
LR test for Block	5	9.03	2	56.11***	4	108.15***	13	40.83***	3	25.96***

*p < .05; **p < .01; ***p < .001

The results show that demographic variables are insignificant in pathological gambling (PG) prediction, while all other groups of variables contribute significantly to the prediction. Among the psychological variables, coping strategies and two gambling motivation types have proven to be significant, for 12 months. Of the variables describing involvement in the game, the game time and, to a lesser extent, money spent on one game session were vital, followed by other e-gambling activities and payments made in pay-to-win games.

These variables were used to build the final predictive model of PG in esports bettors, which was obtained by backward removal of insignificant predictors. Based on the model diagnostics, a scale parameter for the time spent on one ESB session was added. The final model is summarized in Table 5.

**Table 5:** Final predictive model of problem gambling among esports bettors (N = 438)

Type	Parameter	*B*	*SE*	*z*	*OR*	*95%CI[LL, UL]*
Thresholds	1|2	1.15	0.36	3.15		
	2|3	2.46	0.40	6.10		
	3|4	3.82	0.48	8.00		
Location	Escaping coping	0.36	0.09	4.16***	3.15	[1.22. 1.71]
	Engaged coping	− 0.23	0.08	− 2.75**	11.72	[0.67. 0.93]
	Coping motive	0.99	0.14	6.87***	45.49	[2.05. 3.63]
	Financial motive	0.30	0.11	2.71**	1.43	[1.09. 1.69]
	ESB time: linear	0.22	0.21	1.03	0.79	[0.81. 1.91]
	ESB time: quadratic	− 0.38	0.21	− 1.84	2.70	[0.45. 1.04]
	ESB time: cubic	0.50	0.24	2.03*	1.35	[1.00. 2.71]
	ESB time: 4^th^ degree	0.69	0.25	2.72**	1.25	[1.18. 3.35]
	ESB time: 5th degree	0.33	0.23	1.40	0.69	[0.89. 2.27]
	Other e-gambling	0.72	0.17	4.29***	1.65	[1.48. 2.85]
	Play-to-win paying	0.41	0.16	2.59**	1.99	[1.11. 2.07]
Scale	ESB time: linear	− 0.74	0.26	− 2.80**	1.38	[0.29. 0.83]
	ESB time: quadratic	− 0.36	0.24	− 1.50	2.04	[0.43. 1.15]
	ESB time: cubic	− 0.46	0.23	− 1.99*	1.51	[0.40. 1.00]
	ESB time: degree 4	− 0.13	0.21	− 0.63	0.48	[0.57. 1.32]
	ESB time: degree 5	− 0.19	0.19	− 1.01	0.69	[0.58. 1.21]

B, SE, z, OR, LL, and UL represent parameter estimation, with standard error, Wald test statistic, odds ratio, and lower and upper limit of the 95% confidence interval of odds ratio, respectively

*p < .05, **p < .01, ***p < .001

**Fig. 1:**
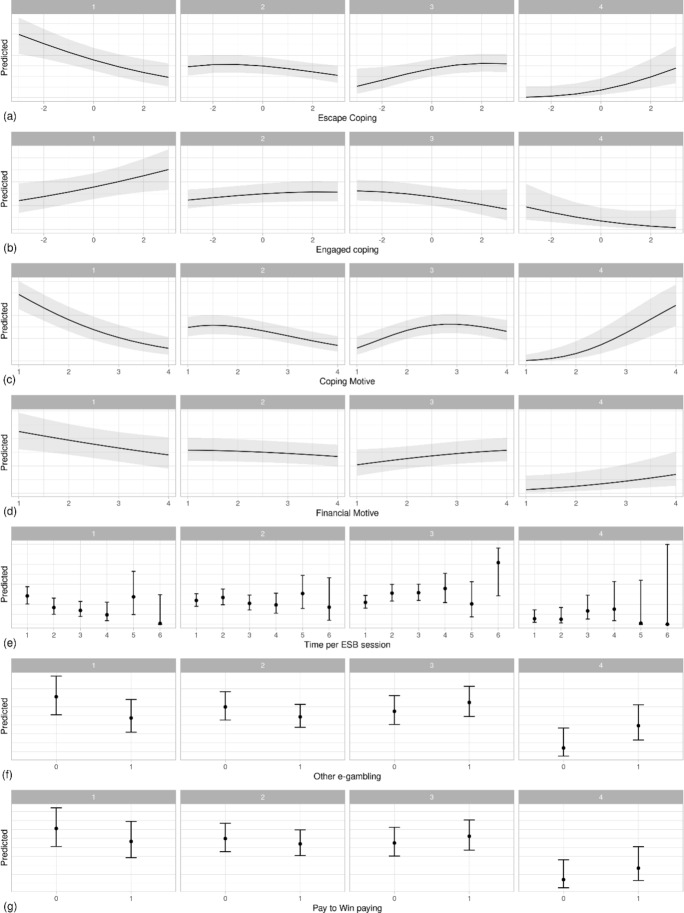
Effect of predictors on the probability of four levels of PG risk among esports bettors (N = 438)

Analysis of the effects of remedial strategies at individual PG risk levels is presented in Fig. 1.

The results of the predictive analysis indicate that coping strategies strongly influence the growing risk of problematic gambling. As the intensity of the escape strategy increases, the probability of being at level 1 (no risk of gambling addiction [from 60 to 20%]) and 2 (low-risk gambling) decreases, while the probability of being at level 3 (moderate risk) or 4 (problem gambling [from 0 to 30%]) increases (Fig. 1a). The opposite is shown to be true for engaged strategies (Fig. 1b): as they intensify, the probability of being at level 1 or 2 of the risk of problem gambling (no risk and low risk) increases, while the probability of being at level 3 and 4 (moderate risk and problem gambling) decreases. The financial and coping motive for gambling work in a similar way (Fig. 1c, d, respectively): as these motives intensify, the probability of being at risk level 1 and 2 decreases, and it increases at risk level 3 and 4, with more substantial dependencies for the coping motive. The time effect shows an interesting, nonlinear relationship. In essence, the effect is linear in the case of low gambling durations. However, at the fifth level of answers (2–3 h spent on a single session of the game), there is a counterintuitive association; this level is characterized by lower average pathogenicity and a much higher confidence interval, which indicates the diversity among people playing very long sessions. Pay-to-win and other forms of e-gambling contribute to a higher risk of problem gambling in esports bettors.

## Discussion

The results of this research study have shown that the factors that best explain an addiction to esports betting are primarily psychological. Problem gamblers use sports betting for the sake of financial gain, as well as to break away from everyday problems and improve their mood. These mechanisms are typical for addicted gamblers engaging in other types of gambling and are confirmed by other studies (MacLaren et al., 2015; Reid et al., 2011). Typical behavior of addicted players is that they refuse to stop playing even when they have lost, which secondarily exacerbates their cognitive distortions associated with belief in winning and consequently perpetuates the adverse gambling behavior leading to addiction (Fortune & Goodie, 2012; Myrseth et al., 2010). Gainsbury drew attention to the risk for both gamers and esports bettors, consisting in the fact that esports bettors often perceive gambling as purely skill games, seeking an analogy to skill-based video games, which increases the risk of their gambling addiction (Gainsbury et al., 2017b). At this point, it is worth noting the phenomenon of professional gaming, in which gaming is treated as a gainful activity. This phenomenon is compared to professional poker, which also carries a risk of addiction for some gamblers, including those who are financially dependent and have low material status (Grzesik, 2019). The search for a way to earn money by gambling with such people will, therefore, be marked by a high risk of a permanent return to gambling and addiction. Research on the relationship between professional gaming and professional gambling has been undertaken, but it is still scarce, and its continuation seems to be very relevant in the context of better understanding of esports and related phenomena (Griffiths, 2017). It is worth noting that the value distribution charts for motivation and coping strategy variables form two patterns (Fig. 1). One is always common for groups 1 and 2, distinguished by their PGSI score (no risk and low risk of problem gambling), and a second pattern is common for groups 3 and 4 (medium risk and problem gambling). This result justifies treating people reaching three or more points in PGSI as one group and explaining the problematic gambling commitment together for these two groups (3–7 and 8 +).

Gambling motivated by the need to cope with difficulties and improve one's mood is strongly linked to unconstructive coping strategies, as confirmed by our research. Those without the ability to actively and constructively deal with difficulties reach for escape strategies, such as stopping activities, taking psychoactive substances, denying, turning to religion, or taking up another activity, as well as esports betting, as was the case in our study group. Escape behavior is typical for people with addictions (Hagström & Kaldo, 2013; Kardefelt-Winther, 2014; Kuss et al., 2012; Li et al., 2011; Ohno, 2016; Xu et al., 2012; Young et al., 2017). It is worth noting that the use of engaged coping strategies acts as a protective factor, decreasing the risk of gambling almost to zero (Fig. 1).

Our results are consistent with research on addiction mechanisms in both gaming disorder and gambling disorder, which suggests that people involved in both activities may exhibit similarities, or that these populations overlap. The results show that as many as 88.6% of bettors also play free online games with the possibility to purchase pay-to-win add-ons, while 59.8% make such purchases in pay-to-win games. This result may indicate a tendency for esports gamblers to spend money on gaming, which is a standard in gambling and, in some way, normalizes this phenomenon in gambling. It can, therefore, be assumed that this type of gambling is a primary activity concerning video games (gaming) and, in particular, about payments made in these games. The normalization of payments made in video games by bettors can be further confirmed by the fact that the dependence on sports betting is significantly related to making payments in pay-to-win games. Players who are addicted to betting also spend larger sums of money on bets than players who are not addicted (result at the significance limit).

Another factor, besides the psychological one, that best explains the addiction to esports betting was the time spent on one esports betting session. Noteworthy at this point are some analogies to gaming disorder. Past research has shown that the time spent on video games alone is not directly related to experiencing the negative consequences of playing (Brunborg et al., 2014). Researchers have pointed out that many video gamers play games with high intensity (often and for a long time), but do not experience significant dysfunctions (Choo et al., 2010; Spekman et al., 2013). An important factor in differentiating these players is the motivation behind their involvement in the games. Treating the game as a way of satisfying one's own needs (a source of need fulfillment) and a way of dealing with stress and frustration proves to be an important risk factor for the development of gaming addiction (Gentile et al., 2011; Loton et al., 2016). For addicted players, gaming is more often a coping strategy than a way to experience entertainment and pleasure (Wan & Chiou, 2006), which is a mechanism consistent with other addictions, including gambling disorder. Escape coping strategies and the motivation to deal with problems through gambling are, therefore, present in both gaming disorder and gambling disorder. However, the emerging difference between the images of both disorders is the intensity of playing. It appears that gamers are more likely to play intensely and without adverse consequences in comparison to gamblers, for whom intensive gambling is most often associated with negative outcomes (Dragicevic et al., 2011; Han et al., 2018). In the case of gambling, the time factor plays a similar role in gaming as in a professional poker game, i.e., one can spend much time in this activity and not experience dysfunction because of it (Griffiths, 2017). In our research, however, despite the linear relationship between time spent on one session of game and betting addiction, a certain deviation was also identified, as shown in Fig. 1e. In groups 1 and 2, separated based on PGSI results, the percentage of people playing in the group decreases with increased in-game time, except for level 5 (2–3 playing hours), where this percentage increases. In the case of groups 3 and 4, on the other hand, the percentage of players increases with increased in-game time, except, again, at level 5 (2–3 h), where the percentage of players decreases. Also, a wider confidence interval indicates a greater variety of players who spend more time on the game. This observation may confirm the phenomenon occurring among video gamers (Griffiths, 2017). It is most likely that there is also a subgroup of bettors (as in the case of e-gamers), which consists of intensive gamblers (intensive from the perspective of the time they spend gambling), but who do not experience any harmful consequences because of this.

Mechanisms similar to other forms of "typical" gambling drive addiction to esports betting, which implies further conclusions. First of all, further research should be devoted to quasi-esports betting, which includes skin gambling, for example. As an unregulated form of entertainment, but governed by the same mechanisms as typical gambling, it should attract the attention of researchers and regulators. Esports seems to be considered more often in the context of sports or computer entertainment, but a significant area of related activity is overlooked, namely the possibility of betting on gaming results. In our research, nearly half of the esports bettors' community also engaged in other forms of online gambling, including slot machines and sports betting, which are considered highly addictive. More than half of the bettors (53.8%), in turn, engaged in offline sports betting. This high engagement rate could suggest a transition from offline gambling to online gambling and then to sports betting and pay-to-win video games. It is worth noting that the percentage of people at risk of becoming addicted to this form of gambling is very high and exceeds 60%, taking into account players at medium and high risk, which suggests a high potential for addiction to this form of gambling. Given that problem gambling is codetermined by different gambling behaviors, but also by gaming behaviors, all types of online gaming behaviors should be taken into account in the problem diagnosis of patients displaying addiction to a particular type of online gambling.

The presented research is one of the few studies devoted to the psychological mechanisms behind the gambling disorder in e-sport bettors. Previous analyses have indicated that blurring the boundaries between gaming and gambling in this type of activity may be related to gambling addiction. Our research has empirically supported the thesis that this blurring in ESB manifests itself in seeing it as a skill-based activity. This is evidenced by the importance of financial motivation, in addition to the motivation to cope expected based on the literature as well as the effect of paying in P2W games. Additionally, this perception of ESB was confirmed by the irrelevance of social motivation, typical to involvement in e-games in general. Our study confirmed that this research line is essential for understanding action addiction mechanisms in esports bettors. The second aspect to which our results contributed is the relationship between the time spent on playing and addiction. The study revealed that in esport bettors, this relationship is nonlinear—people who spend much time on ESB vary significantly in terms of addiction risk.

The results described above raise questions about whether these mechanisms are also crucial in stationary sports betting (for example, through the mechanisms of having expert knowledge in a given field of sport). It seems that in the case of ESB, they may be more robust, as playing games on an advanced level is probably more prevalent among esport bettors than among traditional bettors. Our research results allowed us to formulate these specific hypotheses for future research.

The main limitation of our research is its cross-sectional and not longitudinal design. The direction of the causal relationship cannot be empirically verified (e.g., behavioral playing characteristics may be the result of behavioral disorders and not the other way around).

## Conclusions

Our study has shown that psychological factors, including motivation and coping strategies, have a crucial role to play in explaining problematic esports betting. The activity of esports betting turns out to be connected with other forms of gambling and playing video games, in which one can gain an advantage through payment. The intensity of problematic esports betting increases with the involvement in other types of online games. In the group of people actively involved in intensive esports betting, some do not experience its negative consequences. Engaged coping strategies proved to be a protective factor.
